# An association between individual’s risk perceptions and delayed or foregone healthcare services during the COVID-19 pandemic in Korea

**DOI:** 10.1186/s12913-023-09807-8

**Published:** 2023-08-11

**Authors:** Jongnam Hwang, Woong-Han Kim, Jongho Heo

**Affiliations:** 1 Division of Social Welfare & Health Administration, Wonkwang University, Iksan, Korea; 2 JW LEE Center for Global Medicine, Seoul National University College of Medicine, Seoul, Korea; 3 Department of Thoracic and Cardiovascular Surgery, College of Medicine, Seoul National University, Seoul, Korea; 4 Department of Human Systems Medicine, Seoul National University College of Medicine, Seoul, Korea; 5 National Assembly Futures Institute, Seoul, Korea

**Keywords:** Delayed care, Foregone care, COVID-19, Korea, Healthcare services, Risk perception

## Abstract

**Background:**

Existing evidence highlights that the COVID-19 pandemic is associated with a large reduction in healthcare utilization for routine and less-urgent services around the world including Korea. During the COVID-19 pandemic, delayed and foregone healthcare are driven by various factors, and risk perception, a complex psychological construct, is one of them. The aim of this study was to examine how COVID-19 risk perceptions influence delayed and foregone care during the pandemic in Korea.

**Methods:**

The Koreans’ Happiness Survey (KHS) 2020 was used to analyze responses from 13,491 individuals over 19 years of age residing in Korea. To assess delayed and foregone care, self-reported delayed or foregone care after the COVID-19 outbreak was used. COVID-19 risk perceptions were analyzed in terms of fear and severity of the pandemic based on responses from the participants. Logistic regression models, stratified by gender, were used to examine the relationship between COVID-19 risk perception and delayed/foregone healthcare.

**Results:**

Among the total 13,491 individuals included in the study, 4.0% (n = 541) reported delayed and foregone care in 2020. The results showed that higher level of fear of COVID-19 was associated with an increased likelihood of reporting delayed and foregone care in Korean adults (OR = 1.36, 95% CI = 1.08–1.73). The gender-stratified model revealed that greater fear of COVID-19 was associated with higher odds of delayed and foregone healthcare (OR = 1.71, 95%CI = 1.23–2.39) among women while the perceived severity did not have any association. However, the perceived severity was associated with a higher likelihood of delayed and foregone care in men (OR = 1.17, 95%CI = 1.04–1.32), but no association was found between fear of COVID-19 and delayed and foregone healthcare in men.

**Conclusions:**

To ensure the timely use of any needed healthcare services, it is worth considering establishing policy interventions to mitigate unnecessary fear and worries about COVID-19. This can be achieved by providing accurate information on the virus, protective measures, and treatment.

## Background

The concepts of delayed and foregone health care not only refer to individual situations in which required health services are not provided in a timely manner, but also function as performance indicators that show all types of disruptions in the use of healthcare services [1]. An individual’s reported instance of delayed and foregone health care is the starting point of a vicious cycle that exacerbates the severity of health conditions [2]. Failure to utilize appropriate care services could also be a major cause of the increased use of health services at a later point in time, inducing higher healthcare expenditures [3]. Therefore, understanding the factors associated with delayed and foregone health care is necessary for the development and implementation of health policies to improve the accessibility and use of healthcare services [4].

The ongoing spread of coronavirus disease 2019 (COVID-19) has created unprecedented challenges for healthcare system worldwide [5, 6]. While the full extent of the pandemic’s impact on healthcare services remains unclear, existing evidence suggests that it has led to significant reduction in the use of routine and less-urgent healthcare services in early part of 2020 [7–10]. South Korea (hereafter referred to as Korea) is among the countries that have been successful in controlling COVID-19, with relatively low case and mortality rates due to strong public health measures such as early detection and rapid epidemiological investigations as part of the national response protocol [11]. The Korean government has implemented various policy interventions to ensure all individuals can access healthcare services without COVID-19-related obstacles. These included telephone consultation and prescriptions for all citizens, as well as proxy prescription, which enable someone else to obtain prescription medication on behalf of self-isolated individuals, older adults, and individuals with chronic conditions [12, 13]. Despite these policy interventions aimed at ensuring accessibility of healthcare services [12, 13], a significant decrease in the use of healthcare services was reported in 2020, the first year of the global pandemic. According to a public survey conducted in June 2020, over 65% of the respondents reported a decrease in their visits to clinics and hospitals following the outbreak of COVID-19 in Korea [14]. Furthermore, three out of 10 respondents reported feeling anxious because they were unable to receive necessary health services, and 13% experienced unmet healthcare needs [14]. Studies from other countries with varying incidence rates of COVID-19 have also reported increased proportion of individuals delaying or avoiding healthcare services, suggesting that this is a global phenomenon [15–18].

Delayed and foregone healthcare during the COVID-19 pandemic can be attributed to a wide range of factors. Non-urgent care has been restricted or canceled in many countries due to constraints on resources and hospital capacity [19]. The implementation of strict public measures, such as lockdowns, movement and gathering restrictions, and limited operating hours of healthcare facilities, has also hindered access to necessary healthcare services [20]. Individual-level avoidance or delay, defined as healthcare avoidance, also have played a role in reducing the use of healthcare services for less-urgent care and elective procedures [21]. Furthermore, fear of COVID-19 has also led individuals to hesitate to utilize healthcare services [22]. In fact, numerous studies have suggested a close link between an individual’s health-seeking behavior and their risk perception, which is a complex psychological construct influenced by various factors, such as the immediacy and severity of the risk, and the individual’s knowledge and understanding, emotional well-being and social and cultural factors [23–25]. Risk perception can either facilitate or hamper healthcare utilization by promoting behavioural and lifestyle changes and influencing healthcare decisions. Therefore, it is crucial to understand the impacts of COVID-19-related risk perceptions on delayed and foregone care to develop policies that address obstacles to healthcare during the “living with COVID-19” era. Our aim was to investigate the associations between individual’s risk perceptions and delayed/foregone care during the COVID-19 pandemic in Korea in 2020.

## Methods

### Study design

This study used the Koreans’ Happiness Survey (KHS) 2020, a cross-sectional and national population-based survey that tracks the happiness of Koreans in order to identify policy alternatives to improve the happiness level of the Korean population. The KHS is an annual survey conducted by the National Assembly Future Institutes (NAFI) to monitor the level of happiness and identify various determinants that affect the happiness among the Korean population. The KHS was first initiated in 2020 and has been conducted on an annual basis. The survey collects data on various factors associated with happiness including social and psychological experiences, as well as socio-demographic characteristics. The survey responses were collected using Tablet-Assisted Personal Interviewing (TAPI) method. The interviewer inserted the answers of each respondent into a table immediately during the interview, which allowed for immediate quality checks and minimized non-response. Regarding the COVID-19 module in the KHS, the data was collected only in 2020. The module includes information on the respondents’ healthcare services utilization as well as perceptions, beliefs, experiences, and economic impacts related to the global outbreak. The KHS was conducted among 14,300 people selected from the general Korean population aged 15 and above between October and December 2020, using multi-stage stratified cluster sampling. For this study, 13,491 individuals aged 19 and above who responded to both general and COVID-19 module questionnaires were identified.

### Dependent variable: instances of delayed and foregone care

The dependent variable was the self-reported instances of delayed and foregone care after the COVID-19 outbreak in February 2020. To assess self-reported instances of delayed and foregone care, we used the following questions – “Did you feel the need to receive a medical examination or treatment at hospitals, clinics, or public health centers for purposes other than diagnosis and treatment of COVID-19 after the outbreak of COVID-19(since February 2020)?” The possible responses were “Yes” or “No.” The respondents who reported they needed health care were requested to answer the question, “Did you receive the needed care in a timely manner?” There were three possible response options: “Yes,” “No - received care late,” and “No - gave up.” Based on these two questions, we identified self-reported instances of delayed and foregone care that occurred following the COVID-19 pandemic.

### Independent variable: risk perceptions of COVID-19

Our independent variables of interest were COVID-19 risk perceptions, which were analyzed in terms of fear and severity. These were based on participants’ responses to the following survey questions: “How afraid do you think the current COVID-19 situation?” and “How severe do you think the current COVID-19 situation is?” The possible response options for COVID-19 fear were “very afraid,” “somewhat afraid,” “no feeling about the disease,” “not afraid much” and “not afraid at all,” with the first two collapsed into “fear” and the last three into “no fear” for our analyses. For the question about COVID-19 severity, participants were asked to give a rating ranging from 1 (not severe) to 10 (very severe), and these ratings were analyzed as a continuous variable for our analyses.

### Andersen behavioral model

The Andersen behavioral mode of health services is a conceptual framework that aims to explain and predict the use of individual’s healthcare services [26, 27]. The model suggests that three main factors – predisposing, enabling and need factors- are closely associated with individual’s use of health services as a result of a complex interaction between these three factors. For instance, an individual who perceives a greater need for healthcare services and has access to adequate enabling factors is more likely to use health services than those who limits such resources. The mode has been widely used in the field of health services and policy research to understand healthcare utilization patterns and to identify factors that can improve access to healthcare services.

### Covariate

To examine the associations between COVID-19 risk perceptions and delayed or foregone care, factors that influence the use of healthcare services were selected based on the Anderson health behavior model (HBM) and previous studies [26, 27]. According to the Anderson HBM, factors related to the use of healthcare services are classified into predisposing factors, enabling factors, and need factors [27]. Predisposing factors include demographic characteristics such as age, and enabling factors include socioeconomic status (income and educational levels), residential area, marital status, and participation in economic activities that facilitate healthcare utilization. In the present study, age (i.e.,19–34, 35–49, 50–64, 65+), marital status (i.e., partnered vs. single), and educational attainment(i.e., completion of middle school, high school, and college or above) were included as predisposing factors. Self-reported income, economic activity (i.e., employed, self-employed, non-paid family business, economically inactive) and region (i.e., Seoul metropolitan region vs. non-Seoul metropolitan region) were included as enabling factors. For the need-based factor, a self-rated health status was included in the analytic model.

### Statistical analyses

The possible link between COVID-19 risk perceptions and delayed/foregone healthcare service was examined using logistic regression models after sequential adjustment of risk perceptions and covariates. The first model (Model 1) examined the potential association between individual’s perception of COVID-19 (including fear and severity) and delayed/foregone care among Korean adults. Given that previous research identified gender differences in both perception and healthcare utilization, gender stratified analyses (Model 2 and 3) were conducted [28, 29]. The population weight provided by the KHS was applied to produce estimates representative of the Korean population. Given that correlation between fear and severity of COVID-19 was weak, the association of COVID-19 risk perceptions with delayed and foregone care was assessed in the same analytic model. Data analyses were performed using Stata version 15 (StataCorp LLC, College Station, TX, USA). The results are presented as adjusted odds ratio (AOR) and 95% confidence intervals (95%CI).

## Results

The general characteristics of study participants, stratified by delayed and foregone care, are presented in Table 1. Among the total 13,491 individuals included in the study, 4.0% (n = 541) reported delayed and foregone care in 2020. Older age groups (50–64 and 65+) had higher rates of delayed and foregone care compared to younger age groups (19-34and 35–49). Individuals with fewer than three chronic conditions had the highest reported rate of delayed and foregone care (17.3%), whereas those without any chronic conditions reported the lowest rate (2.9%). Delayed and foregone care was more common among individuals who reported poor self-rated health (10.0%). In addition, respondents who completed high school reported higher rates of delayed and foregone care compared to other educational attainment groups. Individuals dwelling in Seoul metro region reported a higher rate of delayed and foregone healthcare, while a lower rate of delayed and foregone healthcare services was reported among those living in non-Seoul metro region. With respect to COVID-19 perceptions, individuals with a fear of COVID 19 were more likely to report delayed and foregone care.

**Table 1:** General characteristics of study subjects regarding delayed and foregone care– 2020 Koreans’ Happiness Survey (n = 13,491)

Variables			Delayed and foregone care		
		Total (n = 13,491)	Yes – n(%)/mean(SD) (n = 541)	No- n(%)/mean(SD) (n = 12,950)	p-value^*^
Gender	Male	6,399	242 (3.8)	6,157 (96.2)	0.20
	Female	7,092	299 (4.2)	6,793 (95.8)	
Age (years)	19–34	2,777	96 (3.5)	2,681 (96.5)	0.03
	35–49	3,452	121 (3.5)	3,331 (96.5)	
	50–64	5,078	231 (4.5)	4,847 (95.5)	
	65+	2,184	93 (4.3)	2,091 (95.7)	
Chronic conditions	None	11,597	342 (2.9)	11,255 (97.1)	
	Fewer than 3	503	87 (17.3)	416 (82.7)	< 0.05
	3 or more	1,391	112 (8.1)	1,279 (91.9)	
Self-rated health	Good	12,732	465 (3.7)	12,267 (96.3)	< 0.05
	Bad	759	76 (10.0)	683 (90.0)	
Marital status	Partnered	10,071	427 (4.2)	9,644 (95.8)	0.02
	Single	3,420	114 (3.3)	3,306 (96.7)	
Education	Middle school or under	1,427	47 (3.3)	1,380 (96.7)	0.04
	High school	4,697	214 (4.6)	4,483 (95.4)	
	College or above	7,367	280 (3.8)	7,087 (96.2)	
Income	Q1(Lowest)	3,059	116 (3.8)	2,943 (96.2)	0.08
	Q2	3,071	130 (4.2)	2,941 (95.8)	
	Q3	3,519	146 (4.1)	3,373 (95.9)	
	Q4	2,512	113 (4.5)	2,399 (95.5)	
	Q5(Highest)	1,330	36 (2.7)	1,294 (97.3)	
Economic activity	Employed	6,838	271 (4.0)	6,567 (96.0)	0.55
	Self-employed	1,834	82 (4.5)	1,752 (95.5)	
	Non-paid family business	281	14 (5.0)	267 (95.0)	
	Economically inactive	4,538	174 (3.8)	4,364 (96.2)	
Region	Seoul metropolitan region	5,587	353 (6.3)	5,234 (93.7)	< 0.05
	Non-Seoul metropolitan region	7,904	188 (2.4)	7,716 (97.6)	
COVID-19 perceptions					
Fear	Yes	8,999	390 (4.3)	8,609 (95.7)	< 0.05
	No	4,492	151 (3.4)	4,341 (96.6)	
Severity	min 0 ~ max 10		7.17 ± 1.50	7.27 ± 1.45	0.13

^*^ For comparison between delayed/foregone care and no-delayed/foregone care groups, t-test, and chi-square test were conducted accordingly

Table 2 presents the results from logistic regression analyses examining the relationship between individual’s risk perceptions of COVID-19 and delayed/foregone care among Korean adults. The results show higher level of fear of COVID-19 was associated with higher likelihood of reporting delayed and foregone care in Korean adults (Model 1) (OR = 1.36, 95% CI = 1.08–1.73). The gender stratified model revealed that greater fear of COVID-19 was associated with higher odds of delayed and foregone healthcare (OR = 1.71, 95%CI = 1.23–2.39) among women while the perceived severity of COVID-19 did not have any association with delayed and foregone care among them. However, the perceived severity was associated with higher likelihood of delayed and foregone care in men (OR = 1.17, 95%CI = 1.04–1.32). In addition, lower income appeared to be associated with higher likelihood of delayed and foregone care among Korean adults, particularly compared to those in the highest income group. The lowest level of education, completion of middle school or below, was found to be associated with lower likelihood of reporting delayed and foregone care in both women and men (OR = 0.38, 95%CI = 0.21–0.68; OR = 0.44, 95%CI = 0.23–0.87). Moreover, individuals with chronic conditions were more likely to report delayed and foregone care compared to individuals without chronic condition (OR = 6.45, 95%CI = 4.77–8.72; OR = 3.45, 95%CI = 2.59–4.60).

**Table 2:** Relationship between individual’s risk perceptions and delayed/foregone care– 2020 Koreans’ Happiness Survey (n = 13,491)

Variables	Model 1		Model 2		Model 3	
	Overall (n = 13,491)		Female (n = 7,092)		Male (n = 6,399)	
	OR	95%CI	OR	95%CI	OR	95%CI
Perceptions of COVID-19						
Fear	1.36*	1.08–1.73	1.71*	1.23–2.39	1.05	0.75–1.46
Severity	1.05	0.97–1.13	0.96	0.87–1.06	1.17*	1.04–1.32
Gender (ref. male)						
Female	1.08	0.86–1.37				
Age (ref. 19–34)						
35–49	0.75	0.53–1.06	0.71	0.46–1.09	0.81	0.45–1.46
50–64	0.84	0.60–1.18	0.77	0.50–1.17	0.96	0.52–1.75
65+	0.83	0.55–1.26	0.86	0.51–1.47	0.81	0.41–1.62
Chronic condition (ref. 0)						
Fewer than 3	6.45*	4.77–8.72	6.46*	4.30–9.72	6.53*	4.16–10.26
3 or more	3.45*	2.59–4.60	3.62*	2.41–5.44	3.31*	2.20–4.99
Self-rated health (ref. good)						
Bad	1.69*	1.22–2.33	1.89*	1.28–2.78	1.29*	0.73–2.28
Marital status (ref. partnered)						
Single	0.85	0.65–1.13	0.84	0.60–1.19	0.95	0.57–1.59
Education (ref. college or above)						
Middle school or under	0.42*	0.27–0.64	0.38*	0.21–0.68	0.44*	0.23–0.87
High school	0.80	0.63–1.03	0.76	0.55–1.05	0.86	0.58–1.27
Income (ref. Q5)						
Q1 (Low)	1.92*	1.15–3.20	1.43	0.66–3.10	1.75	0.71–4.27
Q2	2.41*	1.50–3.87	1.63	0.72–3.67	3.04*	1.55–5.94
Q3	2.15*	1.39–3.31	1.56	0.67–3.62	2.31*	1.35–3.96
Q4 (High)	2.00*	1.30–3.06	0.96	0.38–2.44	2.49*	1.52–4.07
Economic activity (ref. employed)						
Self-employed	1.13	0.84–1.52	0.83	0.48–1.45	1.28	0.89–1.84
Non-paid family business	1.24	0.69–2.25	1.22	0.61–2.46	1.07	0.27–4.25
Economically inactive	0.92	0.67–1.26	0.87	0.56–1.37	0.93	0.57–1.51
Region (ref. Seoul metropolitan region)						
Non-Seoul metropolitan region	0.30*	0.25–0.38	0.31*	0.24–0.42	0.28*	0.20–0.39

p < 0.05

## Discussion

Using the Koreans’ Happiness Survey (KHS) 2020, a national population-based survey, this study investigated the association between COVID-19 risk perceptions and delayed/foregone care among Korean adults during the global pandemic. Our results revealed that COVID-19 risk perceptions were associated with healthcare service utilization among Korean adults in 2020. In particular, Korean women who had greater fear of COVID-19 infection had higher odds of reporting delayed and foregone care. Moreover, higher perceived severity of COVID-19 was associated with delayed and foregone healthcare services among Korean men, while there was no significant association for Korean women. In addition to risk perceptions, income, and educational attainment, which are well-known determinants of healthcare services utilization, were also found to be related to delayed and foregone care. Meanwhile, the lowest level of educational attainment was associated with a lower likelihood of delayed and foregone care among both women and men.

An individual’s risk perception is a subjective indicator of the physical and social environments and can shape health-seeking behaviors when making health-related decisions [30, 31]. Previous studies have reported that groundless fear and anxiety of COVID-19 could hamper individual’s social activities and have adverse impacts on physical and mental health by causing stress [32, 33]. Similarly, our findings also suggest the pivotal role of COVID-19 risk perceptions as a barrier to access and utilization of necessary healthcare services during the pandemic. We found that higher levels of fear of COVID-19 were associated with a lower likelihood of utilizing necessary healthcare services, which may have significant impacts on disease treatment and management. Our finding implies the need for effective policy strategies to address COVID-19-related fears and ensure the continuity of essential healthcare services. It is also possible that individuals with a greater fear of the virus avoid any risk-taking behaviours, including visiting clinics and hospitals for non-urgent health issues. To ensure the timely provision of any needed healthcare services in the context of the global pandemic crisis in Korea, it is worth considering policy interventions that convey accurate information on the virus, protective measures, and treatment to remedy unnecessary fear and worries about COVID-19.

It is important to note that our study found gender differences in the relationship between COVID-19 risk perceptions and delayed or foregone healthcare services. In particular, we observed that a higher levels of fear of COVID-19 was associated with a higher likelihood of reporting delayed and foregone healthcare services in Korean women, whereas a greater perceived severity of COVID-19 was associated with higher odds of reporting delayed or foregone healthcare services among Korean men. These findings align with previous studies indicating women are more sensitive to the perceived environment and that their health-seeking behaviours are influenced by their perceived fear [34, 35]. It has been suggested that COVID-19risk perceptions should be considered from both cognitive and affective perspectives [36]. Cognitive perception refers to the likelihood of infection and the severity of the disease, while affective risk perception relates to emotional states such as worries, fear, and anxiety of contracting COVID-19 [23, 30]. One plausible explanation of our results is gender differences in responding to risk. Previous studies suggested that women are more likely to report intense emotional responses and negative emotions, while men tended to react more to severity of a situation [37, 38]. In that sense, women who feel more vulnerability or fear in the context of the ongoing pandemic may be more likely to report missed healthcare services, while men who perceive the pandemic as more severe (i.e., with more serious consequences) may be more likely to report delayed or foregone care.

Our study also sheds light on the association of socioeconomic factors with healthcare utilization. Education and income are as well-known determinants of health and healthcare utilization, and we found that they were associated with interruptions in accessing needed healthcare. Prior to the COVID-19 pandemic, it was widely recognized that financial hardship limited the accessibility of needed health services, and income continued to connect pandemic-induced barriers with both delayed and foregone health care [39, 40]. Those with lower income experienced greater difficulties in meeting their healthcare needs, further exacerbating existing inequalities in healthcare, particularly among men. In general, individuals with lower levels of education have higher likelihood of experiencing unmet healthcare needs due to accessibility [40]. However, our results revealed a different pattern during the COVID-19, indicating that individuals with the lowest levels of education encountered fewer healthcare accessibility issues during the COVID-19 pandemic. This could be due to the COVID-19-preventive behaviours that were commonly observed in individuals with higher educational attainment [41]. These individuals might have avoided or canceled healthcare utilization to limit their risk of infection with the virus in the process of receiving health services. In addition, it is possible that individuals with lower levels of education were less aware of their health issues, which led to lower demands and perceived needs for healthcare services.

Furthermore, it is worth noting that the rate of delayed and foregone healthcare services in the KHS was relatively low, at 4% among total respondents, compared to other countries where the frequency ranges from approximately 18 to 50% [20, 42]. The reason for the low rate for delayed and foregone care is unclear, but it is possible that the demand for healthcare services was significantly reduced during the pandemic in 2020, resulting in underreporting of delayed and foregone care. Further studies are needed to fully understand the reasons behind the low rate of delayed and foregone care.

Despite the advantages of this nationally representative data set, which contains various information, this study has several limitations. Given the cross-sectional nature of the Koreans’ Happiness Survey 2020, a limitation of this data set may be that it does not fully incorporate the local and temporal characteristics of the pandemic, which occurred sporadically across local communities and specific population groups in 2020. In addition, information on self-reported instances of delayed and foregone health care was only collected once, which may have led to a failure to fully capture all events of delayed and foregone care in 2020. Delayed and foregone care could arise from different reasons, but the Korean Happiness Survey did not elicit responses about the reasons for delayed and foregone care. It would be worthwhile to collect information on self-reported instances of delayed and foregone care on a regular basis (i.e., each month or wave of the pandemic), as in other countries, and surveys should include a question to identify the causes of delayed and foregone care.

## Conclusions

Our findings showed that fear of COVID-19 increased the likelihood of delayed and foregone care in Korean women while greater perceived severity of COVID-19 in men was associated with higher odds of reporting delayed and foregone care in Korean men. In addition, socioeconomic factors such as lower educational attainment and lower-income level were found to be associated with interruptions in seeking necessary healthcare services. Since timely utilization of healthcare services is crucial, it is essential to establish policy interventions that address unnecessary fears and concerns about COVID-19. This can be achieved by providing accurate information on the virus, public health measures, and treatment.

## Data Availability

The datasets used and analysed during the current study are publicly available in Korea Social Science Data Archive (KOSSDA) repository. https://kossda.snu.ac.kr/handle/20.500.12236/25493.

## List of abbreviations

*KHS*: Koreans’ Happiness Survey
*AOR*: Adjusted Odds Ratio
*TAPI*: Tablet-Assisted Personal Interview
